# An Efficient Near-Field Localization Method of Coherently Distributed Strictly Non-circular Signals

**DOI:** 10.3390/s20185176

**Published:** 2020-09-10

**Authors:** Meidong Kuang, Ling Wang, Yuexian Wang, Jian Xie

**Affiliations:** School of Electronics and Information, Northwestern Polytechnical University, Xi’an 710072, China; meidongkuang628@163.com (M.K.); lingwang@nwpu.edu.cn (L.W.); xiejian@nwpu.edu.cn (J.X.)

**Keywords:** near-filed localization, spacial distributed source, non-circularity, RARE

## Abstract

For the near-field localization of non-circular distributed signals with spacial probability density functions (PDF), a novel algorithm is proposed in this paper. The traditional algorithms dealing with the distributed source are only for the far-field sources, and they need two-dimensional (2D) search or omit the angular spread parameter. As a result, these algorithms are no longer inapplicable for near-filed localization. Hence the near-filed sources that obey a classical probability distribution are studied and the corresponding specific expressions are given, providing merits for the near-field signal localization. Additionally, non-circularity of the incident signal is taken into account in order to improve the estimation accuracy. For the steering vector of spatially distributed signals, we first give an approximate expression in a non-integral form, and it provides the possibility of separating the parameters to be estimated from the spatially discrete parameters of the signal. Next, based on the rank-reduced (RARE) algorithm, direction of arrival (DOA) and range can be obtained through two one-dimensional (1-D) searches separately, and thus the computational complexity of the proposed algorithm is reduced significantly, and improvements to estimation accuracy and identifiability are achieved, compared with other existing algorithms. Finally, the effectiveness of the algorithm is verified by simulation.

## 1. Introduction

Source localization is an important branch in the field of array signal processing, and significant achievements have been made in this field in recent decades [1,2]. However, most of the previous studies focused on far-field signal parameter estimation [3,4]. On the other hand, research on near-field source localization, which requires estimation of both DOA and the range of the signals has received increasing attention [5]. The traditional DOA estimation algorithms for far-field signals are no longer suitable for near-field sources [6]; thus, many scholars have proposed parameter estimation algorithms for near-field models in recent years, most of which are based on higher-order statistics (HOS) [7,8]. These algorithms can mitigate Gaussian colored noise well [7], at the cost of a substantial increase in calculations. Hence, lowering the computational complexity of the near-field localization, which is arising as an important research issue, has been investigated, especially in the context of second-order statistics. In [8], Li et al. developed a computationally efficient near-field localization method with simplified fourth-order cumulants. However, it still needs two-dimensional spectral peak searches, and the error caused by Schmidt’s orthogonalization therein will seriously affect the range estimation. However, compared with methods using higher-order statistics, these SOS-based algorithms have been at a disadvantage in estimation accuracy. Classical second-order statistics algorithms include the weighted linear prediction method [9] and the generalized ESPRIT (GESPRIT)-based method [10].

To further improve estimation accuracy, much effort has been devoted to exploiting the structure of the signal, such as non-circular characteristics [11]. As is widely known, non-circularity is a universal characteristic of communication signals, such as in binary phase shift keying (BPSK), offset quadrature phase shift keying (OQPSK), and pulse amplitude modulation (PAM). It has been revealed that non-circularity can greatly enhance both the estimation accuracy and identifiability, resulting in novel research findings. The MUSIC-like algorithm for non-circular sources was first proposed in 1998 by [12]. Romer et al. tailored the ESPRIT method to non-circular signals and the corresponding Cramer–Rao lower bound (CRLB) in [13] was derived to show the superiority of the algorithm. In [14], the author provided a non-circular asymptotically minimum variance algorithm and the estimation performance was close to CRLB, while requiring a very large amount of calculations due to the multi-dimensional search. In [15,16], the CRLB for the non-circular signals in color or in Gaussian white noise were discussed separately. An improved MUSIC algorithm was proposed with a more general scenario where both the circular and non-circular sources coexist [17]. The idea based on sparse representation was extended to non-circular cases in [18]. A two-dimensional (2D) DOA estimation of non-circular sources was proposed in [19]. Unfortunately, the algorithms described above are only applicable to far-field models. To the best of our knowledge, there are few localization algorithms that study near-field non-circular signals, except for the work in [20], where Xie et al. utilized the advantages of non-circular signals to improve localization performance, and then in the follow up work [21], the same authors considered a more complex scenario where mixed far-field and near-field sources impinge on the array with unknown mutual coupling.

On the other hand, most algorithms assume that far-field signals are point sources, and assume so also for near-field signals; this cannot be utilized in many practical applications [22], especially for near-filed localization where the angular expansion caused by the movement of the target in space cannot be ignored. Compared to far-field targets, near-field sources are much closer to the sensor array. For far-field signals, the angles may hardly change, while for near-field signals, the angle dithering can be more dramatic. However, little research has been done on this issue so far. Jantti et al. proposed to treat distributed sources as a set of point sources and then used traditional MUSIC and ESPRIT algorithms to determine the DOA parameter. Although the method is workable, one target requires many more degrees of freedom than the point source case for parameter estimation, leading to a larger size, higher complexity and higher cost of the sensor array [23]. Besides, it is difficult or even impossible to know what the distribution law of the target is in advance. The maximum likelihood method was also used to solve the problem of distributed source localization in [24]. The computational complexity of maximizing the likelihood function increased exponentially due to the introduction of distribution parameters. Given the parameterized shape of the distribution, the authors in [25] attempted to use discrete modeling methods to deal with non-overlapping sources. In [26], Chaaya et al. attempted to localize and characterize coherently distributed (CD) sources in the near-field range. However, for the range estimation, they supposed the signals are point sources, which means the spatial distribution characteristics of signals was ignored, causing inherent error. The same problem occurred in [2], in which Wan et al. only considered the special condition but ignored source spatial distribution although circular and non-circular sources were mixed together.

The aforementioned reasons motivated us to propose an estimator of CD sources in the near-field that is robust enough to accommodate the imperfect knowledge of the angular spread distribution. Thus, in this paper we provide a method that can get the angle and range estimation while eliminating the influence of angular spread distribution. In the new model, we relax the constraints on the spatial distribution, that is, the probability density distribution of angle is known a priori but the specific parameters are unknown. In order to improve estimation performance, the non-circular property of signals is considered, and a rank reduction (RARE) method for non-circular CD sources (NCSD-RARE) is proposed. It can effectively separate the DOA and range parameters form the non-circular parameters needed for applying the RARE principle, which only needs two 1D searches; hence the influence arising from the unfixed state of the spatial distribution can be mitigated in this method.

## 2. Problem Formulation

### 2.1. Signal Model

**Figure 1:**
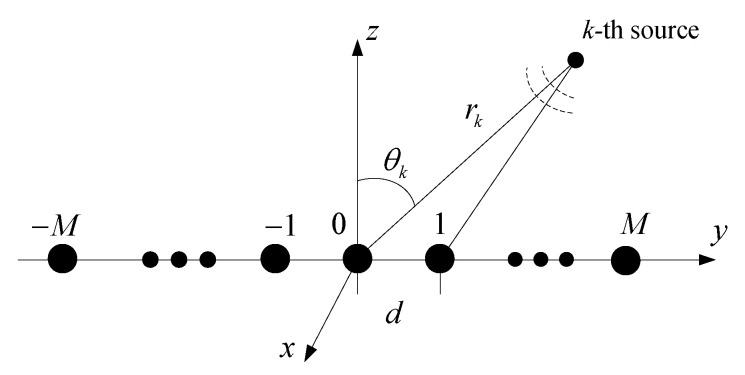
The near-field source with the symmetric ULA.

Consider a symmetrical uniform linear array (ULA) with 2M+1 sensors; the spacing between its elements is set to *d*, and it does not exceed one quarter of the wavelength of the received signal to avoid phase ambiguity. K<(2M+1) uncorrelated narrow-band CD non-circular sources are received by the array, as shown in Figure 1. The data received by the 2M + 1 sensors can be expressed as $x\left(t\right)={\left[x_{-M}\left(t\right),\ldots ,x_{0}\left(t\right),\ldots ,x_{M}\left(t\right)\right]}^{T}$. Considering an angular spread of DOA, one has:

(1) $$\begin{array}{c} x\left(t\right)=\sum_{i=-M}^{M}\int_{-\pi /2}^{\pi /2}a(\theta ,r_{i})v_{i}\left(\theta ,\theta_{i},\Delta_{i},t\right)d\theta +n\left(t\right), \end{array}$$

where $v_{i}\left(\theta ,\theta_{i},\Delta_{i},t\right)$ is the signal angular distribution of the *i*-th source, $\Delta_{i}$ is the angular spread, and $\theta_{i}$ is the central DOA for the source. n(t) is a vector expression of Gaussian white noise caused by the system. The steering vector a(ϕ,r) represents the near-field array response, which is related to the DOA ϕ and the range *r*. Therefore, observations received by the array at time *t* are as follows:

(2) $$\begin{array}{c} x(t)=\mathrm{Bs}(t)+n(t), \end{array}$$

where $B=[b_{-M},\ldots ,b_{0},\ldots ,b{}_{M}]$ is the (2M+1)×K generalized array manifold, $s\left(t\right)={\left[s_{1}\left(t\right),\ldots ,s_{k}\left(t\right),\ldots ,s_{K}\left(t\right)\right]}^{T}$ is the K×1 signal vector, and n(t) is the AWGN vector with zero means and variance $\sigma_{n}^{2}$. $b(\theta_{i},r_{i}.\Delta_{i})$ is given by:

(3) $$\begin{array}{c} b\left(\theta_{i},r_{i},\Delta_{i}\right)=\int_{-\pi /2}^{\pi /2}a(\theta_{i},r_{i})g_{i}\left(\theta ,\theta_{i},\Delta_{i}\right)d\theta . \end{array}$$

Thus $b(\theta_{i},r_{i},\Delta_{i})$ is the vector accommodating the steering vector $a(\theta_{i},r_{i})$ with the angular distribution of the *i*-th source with the probability density function (PDF) $g_{i}\left(\theta ,\theta_{i},\Delta_{i}\right)$. Then, we define $\tau_{mk}$ as the time delay of the 0-th sensor and the *m*-th sensor when receiving the *k*-th signal:

(4) $$\begin{array}{c} \tau_{mk}=\frac{2\pi }{\lambda }\left(\sqrt{r_{k}^{2}+{\left(md\right)}^{2}-2r_{k}md\sin\theta_{k}}-r_{k}\right), \end{array}$$

where $\theta_{k}$ ,$r_{k}$, and λ respectively represent the azimuth, distance, and wavelength of the *k*-th signal. The limiting condition for wavelength λ is λ≥4d. The phase difference can be expressed as follows via second-order Taylor expansion:

(5) τmk=(−2πdλsinθk)m+(d2λrkcos2θk)m2+o(d2d2rk2rk2)≈ωkm+ϕkm2.

In the formula above,

(6) $$\begin{array}{c} \omega_{k}=-2\pi \frac{d}{\lambda }\sin\theta_{k}, \end{array}$$

(7) $$\begin{array}{c} \phi_{k}=\pi \frac{d^{2}}{\lambda r_{k}}\cos^{2}\theta_{k}. \end{array}$$

Hence $a(\theta_{i},r_{i})$ has the following expression:

(8) $$\begin{array}{c} A=[a\left(\omega_{1},\phi_{1}\right),a\left(\omega_{2},\phi_{2}\right),\ldots ,a\left(\omega_{K-1},\phi_{K-1}\right),a\left(\omega_{K},\phi_{K}\right)]. \end{array}$$

For the 1-D signal model of a CD non-circular source, the PDF of angular spread $g_{i}\left(\theta ,\theta_{i},\Delta_{i}\right)$ could be uniform distribution, triangular distribution, or Gaussian distribution.

### 2.2. Proposed Method

From (3), we defined:

(9) $$\begin{array}{c} \beta_{k}\left(\mu \right)={\left[b\left(\beta ,r,\Delta \right)\right]}_{k}\approx {\left[a\left(\beta ,r\right)\right]}_{k}g{\left[\theta ,\Delta \right]}_{k}, \end{array}$$

where ${\left[a\left(\beta ,r\right)\right]}_{k}$ is the complex exponential term defined as:

(10) $$\begin{array}{c} {\left[a\left(\beta ,r\right)\right]}_{k}=\exp\left(j\times (\omega_{k}+\phi_{k})\right). \end{array}$$

Since the PDF of the angular spread is symmetrical to θ, $g{\left[\theta ,\Delta \right]}_{k}$ is a real number, *k* = 1,2, *…*, *M*. When the CD source follows a uniform distribution, the PDF of the angular spread is given by:

(11) $$\begin{array}{c} g_{i}\left(\theta ,\theta_{i},\Delta_{i}\right)=\left\{\begin{array}{c} \frac{1}{2\Delta_{i}},\left|\theta -\theta_{i}\right|\leq \Delta_{i}\\ 0,\left|\theta -\theta_{i}\right|>\Delta_{i} \end{array}\right., \end{array}$$

where $\theta_{i}$ is the angular range of the different incident signals in one CD source. The corresponding ${\left[g\left[\theta ,\Delta \right]\right]}_{k}$ becomes:

(12) $$\begin{array}{c} {\left[g\left[\theta ,\Delta \right]\right]}_{k}=\frac{\sin\left(-\left(k-1\right)\right)\Delta_{i}}{-\left(k-1\right)\Delta_{i}}. \end{array}$$

If the CD source obeys the Gaussian distribution, then the PDF of the angular spread is given by:

(13) $$\begin{array}{c} g_{i}\left(\theta ,\theta_{i},\Delta_{i}\right)=\frac{1}{\sqrt{2\pi \Delta_{i}^{2}}}\exp\left(-\frac{{\left(\theta -\theta_{i}\right)}^{2}}{2\Delta_{i}^{2}}\right). \end{array}$$

The corresponding ${\left[g\left[\theta ,\Delta \right]\right]}_{k}$ is given as:

(14) $$\begin{array}{c} {\left[g\left[\theta ,\Delta \right]\right]}_{k}=e^{M^{2}\Delta_{i}^{2}/2}. \end{array}$$

For the triangular distribution, the corresponding PDFs of the angular spread and ${\left[g\left[\theta ,\Delta \right]\right]}_{k}$ are, respectively:

(15) $$\begin{array}{c} g_{i}\left(\theta ,\theta_{i},\Delta_{i}\right)=\left\{\begin{array}{cc} \left(\theta -\theta_{i}+\Delta_{i}\right)/\Delta_{i}^{2} & -\Delta_{i}\leq \theta -\theta_{i}<0\\ \left(-\theta +\theta_{i}+\Delta_{i}\right)/\Delta_{i}^{2} & 0\leq \theta -\theta_{i}\leq \Delta_{i}\\ 0 & else \end{array}\right. \end{array}$$

and

(16) $$\begin{array}{c} {\left[g\left[\theta ,\Delta \right]\right]}_{k}=\frac{2[1-\cos\left(M\right)\Delta_{i}]}{M^{2}\Delta_{i}^{2}}. \end{array}$$

As a result, we obtain the following steering vector for the three kinds of the angular spread PDFs. For the uniform distribution, the steering vector is:

(17) $$\begin{array}{c} \begin{array}{c} b\left(\theta_{i},r_{i},\Delta_{i},\right)=[\frac{\sin\left(-M\right)\Delta_{i}}{-M\Delta_{i}}e^{j(-M\omega +{\left(-M\right)}^{2}\phi )},\frac{\sin\left(-\left(M-1\right)\right)\Delta_{i}}{-\left(M-1\right)\Delta_{i}}e^{j(-\left(M-1\right)\omega +{\left(-(M-1)\right)}^{2}\phi )},\ldots ,1,\\ \ldots ,\frac{\sin\left(M-1\right)\Delta_{i}}{\left(M-1\right)\Delta_{i}}e^{j(\left(M-1\right)\omega +{\left((M-1)\right)}^{2}\phi )},\frac{\sin\left(M\right)\Delta_{i}}{M\Delta_{i}}e^{j(M\omega +{\left(M\right)}^{2}\phi )}] \end{array}. \end{array}$$

For the Gaussian distribution, the steering vector is:

(18) $$\begin{array}{c} \begin{array}{c} b\left(\theta_{i},r_{i},\Delta_{i}\right)=[e^{-M^{2}\Delta_{i}^{2}/2}e^{j(-M\omega +{\left(-M\right)}^{2}\phi )},e^{{\left(-(M-1)\right)}^{2}\Delta_{i}^{2}/2}e^{j(-\left(M-1\right)\omega +{\left(-(M-1)\right)}^{2}\phi )},\ldots ,1,\\ \ldots ,e^{{\left(M-1\right)}^{2}\Delta_{i}^{2}/2}e^{j(\left(M-1\right)\omega +{\left((M-1)\right)}^{2}\phi )},e^{M^{2}\Delta_{i}^{2}/2}e^{j(M\omega +{\left(M\right)}^{2}\phi )}] \end{array}. \end{array}$$

For the triangular distribution, the steering vector is:

(19) $$\begin{array}{c} b\left(\theta_{i},r_{i},\Delta_{i},\right)=[\frac{2[1-\cos\left(-M\right)\Delta_{i}]}{-M^{2}\Delta_{i}^{2}}e^{j(-M\omega +{\left(-M\right)}^{2}\phi )},\frac{2[1-\cos\left(-\left(M-1\right)\right)\Delta_{i}]}{-{\left(M-1\right)}^{2}\Delta_{i}^{2}}e^{j(-\left(M-1\right)\omega +{\left(-(M-1)\right)}^{2}\phi )},\ldots ,1,\\ \ldots \frac{2[1-\cos\left(M-1\right)\Delta_{i}]}{{\left(M-1\right)}^{2}\Delta_{i}^{2}}e^{j(\left(M-1\right)\omega +{\left((M-1)\right)}^{2}\phi )},\frac{2[1-\cos\left(M\right)\Delta_{i}]}{M^{2}\Delta_{i}^{2}}e^{j(M\omega +{\left(M\right)}^{2}\phi )}] \end{array}.$$

**Figure 2:**
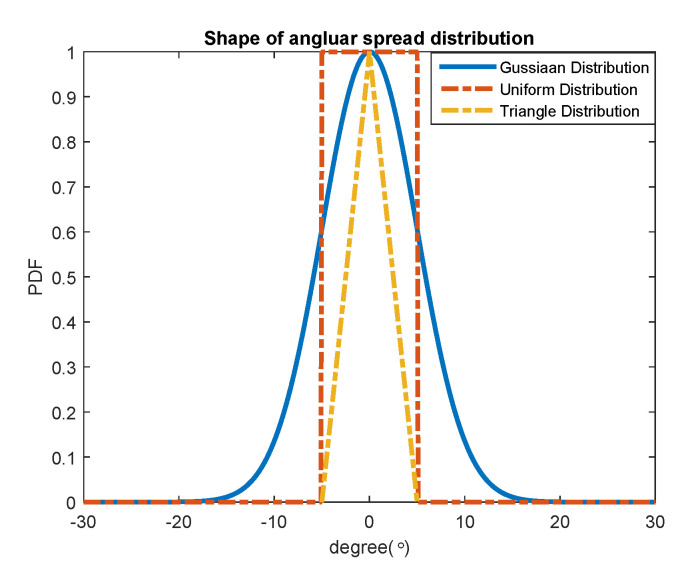
Three classic shapes of the angular spread distribution.

Next we define ℓ(M,Δ) as follows:

(20) $$\begin{array}{c} \ell \left(M,\Delta \right)=\left\{\begin{array}{ccc} e^{-M^{2}\Delta^{2}/2} & Gaussian & \\ \frac{\sin(M)\Delta }{M\Delta } & uniform & \\ \frac{2[1-\cos(M)\Delta ]}{M^{2}\Delta^{2}} & triangular & \end{array}\right., \end{array}$$

and the generalized steering vector b(θ,r,Δ) can be factorized as:

(21) $$\begin{array}{c} b(\theta ,r,\Delta )=l⊙A, \end{array}$$

where l is the (2M+1)×1 vector containing the entry ℓ(M,Δ). ⨀ is the Hadamard–Schur matrix. These three classic shapes of the angular spread distribution are shown in Figure 2.

### 2.3. NCSD-RARE Method

Extracting the non-circularity embedded into the signal waveform $S\left(t\right)$, the transmitted signal can be factorized as [20]:

(22) $$\begin{array}{c} S\left(t\right)=\Psi^{1/2}S_{0}\left(t\right), \end{array}$$

where $S_{0}\left(t\right)={\left[s_{0,1}\left(t\right),\ldots ,s_{0,K}\left(t\right)\right]}^{T}\in R^{K\times 1}$, $s_{0,k}\left(t\right)$ is the real symbol of $s_{k}\left(t\right)$, and $\Psi^{1/2}=diag\left\{e^{j\varphi_{1}/2},\ldots {,}^{j\varphi_{K}/2}\right\}$ contains the non-circular phases on the diagonal.

We take the non-circular property of sources into account, and have the following argument data matrix:

(23) $$\begin{array}{c} Y\left(t\right)=\left[\begin{array}{c} X(t)\\ X^{*}\left(t\right) \end{array}\right]=\left[\begin{array}{c} B\\ B^{*}\Psi^{*} \end{array}\right]S_{0}\left(t\right)+\left[\begin{array}{c} N\left(t\right)\\ N^{*}\left(t\right) \end{array}\right], \end{array}$$

where $B=[b\left(\theta_{1},r_{1},\Delta_{1},\varphi_{1}\right),\ldots ,b\left(\theta_{K},r_{K},\Delta_{K},\varphi_{K}\right)]$ is the array manifold of the spacial distributed signals with the following augmented near-field steering vector as:

(24) $$\begin{array}{c} \bar{b}\left(\theta ,r,\Psi ,\Delta \right)=\left[\begin{array}{c} b(\theta ,r,\Delta )\\ b^{*}\left(\theta ,r,\Delta \right)e^{-j\Psi } \end{array}\right], \end{array}$$

then the observation vector can be rewritten in a matrix format as:

(25) $$\begin{array}{c} Y\left(t\right)=\bar{B}S_{0}\left(t\right)+\bar{N}\left(t\right). \end{array}$$

Performing eigen-decomposition, the augmented data covariance matrix R is separated to:

(26) $$\begin{array}{c} R=E\left\{Y\left(t\right)Y^{H}\left(t\right)\right\}=U_{S}\Lambda_{S}U_{S}^{H}+U_{N}\Lambda_{N}U_{N}^{H}, \end{array}$$

where $\Lambda_{S}$ is a diagonal matrix, corresponding to the *K* largest eigenvalues in the original covariance matrix **R**. Similarly, the other small eigenvalues in matrix **R** correspond to the matrix $\Lambda_{N}$. According to the subspace theory, the augmented array manifold $\bar{b}$ and the eigenvector matrix $U_{S}$ share the same space, and the subspace spanned by the eigenvector matrix $U_{N}$ is the noise subspace. Based on the assumption that signal and noise are uncorrelated to each other, using the following function to traverse the maximum energy within a certain angle range, one can obtain the estimate of the angle:

(27) $$\begin{array}{c} P\left(\theta ,r,\Psi ,\Delta \right)={\bar{b}}^{H}\left(\theta ,r,\Psi ,\Delta \right)U_{N}U_{N}^{H}\bar{b}\left(\theta ,r,\Psi ,\Delta \right). \end{array}$$

However, in order to find the minimum value of the spectral peak, (27) requires a four-dimensional (4D) search, bringing about an impractical calculation load. To reduce the amount of computation, we first decouple the DOA from the other three parameters by the principle of RARE, and obtain the DOA through one-dimensional search. Because the ULA is symmetrical about the center of its array, the augmented steering vector in (27) can be reparameterized as:

(28) $$\begin{array}{c} \bar{b}\left(\theta ,r,\Psi ,\Delta \right)=\underset{\bar{\Gamma }}{\underset{︸}{\left[\begin{array}{cc} \Gamma (\theta ) & 0\\ 0 & \Gamma^{*}\left(\theta \right) \end{array}\right]}}\underset{\bar{h}}{\underset{︸}{\left[\begin{array}{c} h(\phi ,\Delta )\\ h^{*}\left(\phi ,\Delta \right)e^{-j\Psi } \end{array}\right]}}. \end{array}$$

Since the columns of $U_{S}$ and $\bar{b}\left(\theta ,r,\Psi ,\Delta \right)$ are orthogonal, the following formula can be derived:

(29) $$\begin{array}{c} {\bar{b}}^{H}\left(\theta_{k},r_{k},\Psi_{k},\Delta_{k}\right)U_{N}U_{N}^{H}\bar{b}\left(\theta_{k},r_{k},\Psi_{k},\Delta_{k}\right)=0,\;k=1,\cdots ,K. \end{array}$$

Substituting (28) into (29), we have:

(30) $$\begin{array}{c} {\bar{h}}^{H}\left(\theta ,r,\Psi ,\Delta \right)\underset{ℵ\left(\theta \right)}{\underset{︸}{{\bar{\Gamma }}^{H}\left(\theta \right)U_{N}U_{N}^{H}\bar{\Gamma }\left(\theta \right)}}\bar{h}\left(\theta ,r,\Psi ,\Delta \right)=0. \end{array}$$

Obviously $ℵ\left(\theta \right)$ is not an all-zero matrix. According to the idea of rank reduction, the (30) can be equivalent to:

(31) $$\begin{array}{c} \underset{ℵ\left(\theta \right)}{\underset{︸}{{\bar{\Gamma }}^{H}\left(\theta \right)U_{N}U_{N}^{H}\bar{\Gamma }\left(\theta \right)}}=0. \end{array}$$

According to the idea of the MUSIC algorithm, we can obtain the angle of the target through 1D spectral peak search.

(32) $$\begin{array}{c} P_{1}\left(\theta \right)=\frac{1}{\det\{ℵ(\theta )\}}. \end{array}$$

Through a similar approach, $\bar{b}\left(\theta ,r,\Psi ,\Delta \right)$ can be also decoupled as:

(33) $$\begin{array}{c} \bar{b}\left(\theta ,r,\Psi ,\Delta \right)=\underset{\bar{H}}{\underset{︸}{\left[\begin{array}{cc} a(\theta ,r) & 0\\ 0 & a^{*}\left(\theta ,r\right) \end{array}\right]}}\underset{q}{\underset{︸}{\left[\begin{array}{c} e^{M^{2}\Delta }\\ e^{-j\Psi -M^{2}\Delta } \end{array}\right]}}. \end{array}$$

Substituting (33) into (27), we have:

(34) $$\begin{array}{c} q^{H}\left(\Psi ,\Delta \right)\underset{\Xi \left(\theta ,r\right)}{\underset{︸}{{\bar{H}}^{H}\left(\theta ,r\right)U_{N}U_{N}^{H}\bar{H}\left(\theta ,r\right)}}q\left(\Psi ,\Delta \right)=0. \end{array}$$

By the same principle, Ξ(θ,r) is not an all-zero matrix. According to the principle of RARE, the rank of Ξ(θ,r) drops if and only if $\theta =\theta_{k}$ and $r=r_{k}$, where k=1,⋯,K. Therefore, with the DOA estimates $\theta_{k}$ obtained from (32), the range parameters can be generated by substituting each $\theta_{k}$ back into the following RARE estimator:

(35) $$\begin{array}{c} P_{2}^{\left(k\right)}\left(r\right)=\frac{1}{\det\left\{\Xi \left({\hat{\theta }}_{k},r\right)\right\}},\;k=1,\cdots ,K\left(\theta =\theta_{k}\right). \end{array}$$

As can be seen from (35), the angle and range can be automatically paired without any other operation. Additionally, it can be seen that the maximum number of sources that can be estimated by this algorithm is K⩽2M. Compared to traditional second-order statistics algorithms, the identification capability of the proposed method has been doubled. In addition, the estimation accuracy of the proposed algorithm is also greatly improved, which will be verified through the following simulations.

## 3. Simulation Results and Discussion

In this section, the performance of the proposed algorithm will be tested under different experimental conditions, and a comparison will be conducted between them. Assume near-field signals are received by a ULA with a number of array elements of N = 7 (M = 3), where the spacing element *d* is a quarter of the wavelength of the received signal. The received signal is set to the common BPSK, typically non-circular signal. Performance is measured using root mean square error (RMSE), which is defined as:

(36) $$\begin{array}{c} RMSE=\sqrt{\frac{1}{500}\sum_{n=1}^{500}{\left({\hat{z}}_{n,k}-z_{k}\right)}^{2}}. \end{array}$$

Among them, $z_{k}$ represents the parameters to be counted, and in this article refers to the DOA range. ${\hat{z}}_{k}$ is the estimate in the *k* experiment and 500 refers to the number of experiments repeated under the same conditions to ensure that the statistics are close to the theory.

**Figure 3:**
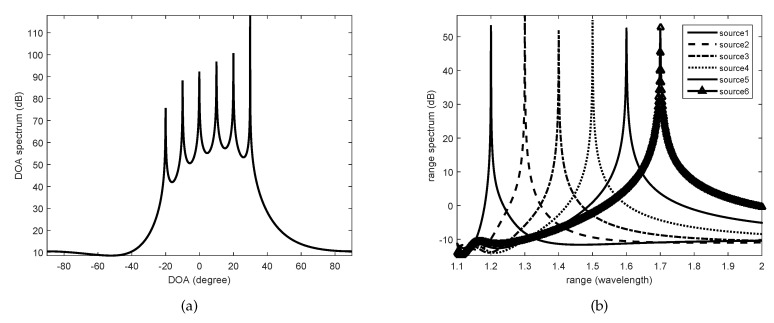
The spatial spectrum of DOA and range of six uncorrelated signals complying with Gaussian distribution of angular spread when SNR=15 dB. (**a**) The spatial spectrum of DOA of the six uncorrelated signals, (**b**) The spatial spectrum of range of the six uncorrelated signals with each estimated DOA.

In the first experiment, we considered six BPSK sources. Their position information relative to the reference element was as follows: $\left(-20^{\circ },1.2\lambda \right)$, $\left(-10^{\circ },1.3\lambda \right)$, $\left(0^{\circ },1.4\lambda \right)$, $\left(10^{\circ },1.5\lambda \right)$, $\left(20^{\circ },1.6\lambda \right)$, and $\left(30^{\circ },1.7\lambda \right)$. In addition, they all obeyed the Gaussian distribution, and the corresponding spatially distributed parameters were $1^{\circ }$, $3.3^{\circ }$, $2^{\circ }$, $3^{\circ }$, $2.4^{\circ }$, and $2.5^{\circ }$, respectively. The SNR was set as 15 dB. The DOA and range spectra are shown in Figure 3. It can be seen that in the spectrum of the DOA, six peaks can be clearly identified. With the angle information that we have obtained, we can see six corresponding distance spectrum peaks in turn.

**Figure 4:**
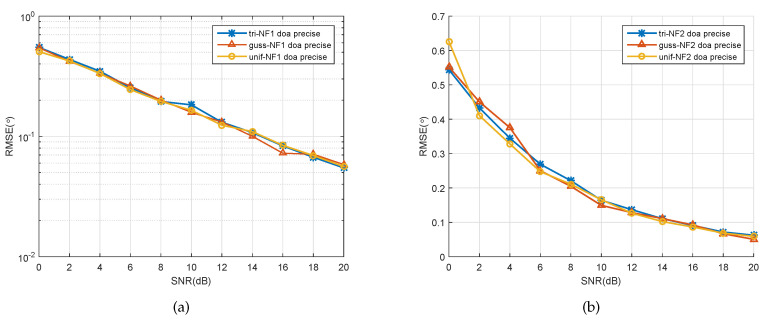
RMSE of the DOA estimates of two signals versus SNR. (**a**) First spatially distributed source, (**b**) second spatially distributed source.

In the second scenario, we considered two sources following one of the distributions (Gaussian, uniform, or triangle) from $\left(-20^{\circ },1.2\lambda \right)$ and $\left(-10^{\circ },1.3\lambda \right)$ that impinge on a five-element ULA, of whose spatially distributed parameters’ Δ are $1^{\circ }$ and $3.3^{\circ }$, respectively. The number of snapshots was set to 200, and the SNR varied from 0 to 20 dB in a step-size of 2 dB. It is clear that the accuracy of the DOA estimates of the first spatially distributed signal increased with the growth of SNR, as shown in Figure 4a. For the second spatially distributed signal estimation, the results indicated a similar conclusion, as seen in Figure 4b. At the same time, we can still see a small difference between the two sources with three kinds of PDF, that is, a steady decline in the estimation errors of the second distributed signal with the growth of SNRs. This is because that the estimation accuracy is reliant upon the spatial distribution parameter ∇, which will be further verified in following simulations.

**Figure 5:**
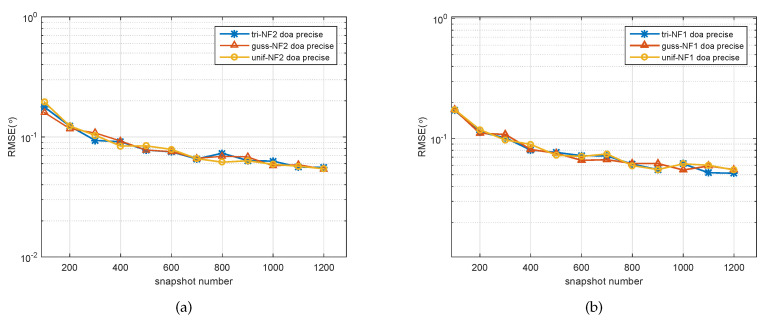
RMSE of the DOA estimates of two signals versus snapshots. (**a**) First spatially distributed source with three kinds of PDF, (**b**) second spatially distributed with three kinds of PDF.

In the third simulation, the relationship of the parameter estimation accuracy with the snapshot number was studied. We considered distributed signals from $\left(-20^{\circ },1.2\lambda \right)$ and $\left(-10^{\circ },1.3\lambda \right)$ impinging on a five-element ULA, whose spatially distributed parameters’ Δ were $1^{\circ }$ and $3.3^{\circ }$, respectively. The SNR was set to 15 dB, and the number of snapshots varied from 100 to 1200 in a step-size of 100. As seen in Figure 5a, the RMSEs of the DOA estimates descended in a steady trend with the increase of the number of snapshots. The results indicated a similar conclusion for the second source, as seen in Figure 5b.

**Figure 6:**
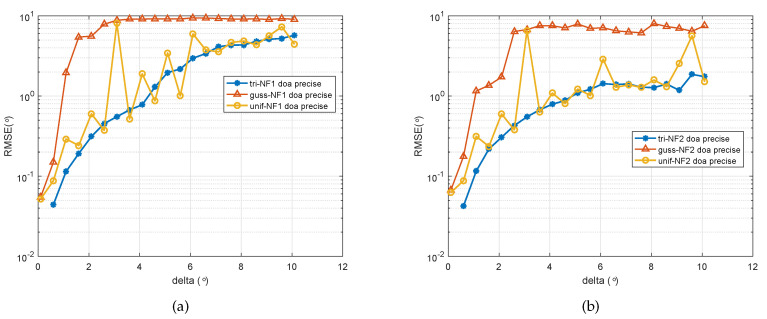
RMSE of the DOA estimates of two signals versus spatial distribution parameter ∇. (**a**) First spatially distributed source with three types of PDF, (**b**) second spatially distributed source with three types of PDF.

In the final experiment, the relationship of the parameter estimation accuracy with the spatially distribution was showed. We considered two distributed sources from $\left(-20^{\circ },1.2\lambda \right)$ and $\left(-20^{\circ },1.2\lambda \right)$ impinging on a five-element ULA. The SNR was set to 15 dB and the number of snapshots was set as 200. The spatially distributed parameters’ Δ varied from 0.1 to 10 in a step-size of 0.5. As can be seen in Figure 6a, the DOA estimation error of two signals became bigger as the spatially distributed parameter Δ growed, which means that the amplitude of estimated target shaking was bigger, making it more difficult to ascertain the location of the target. This also supports an explanation of the difference between Figure 4a,b. It can be observed that the estimation performance for the second source under all three spatial distributions became worse in general. Alternatively, we can compare three signals with the different kinds of PDF. It is obvious that the signal following Gaussian distribution was more sensitive to the spatial spread parameter, and the signal following a uniform distribution changed more erratically compared with the two others.

**Figure 7:**
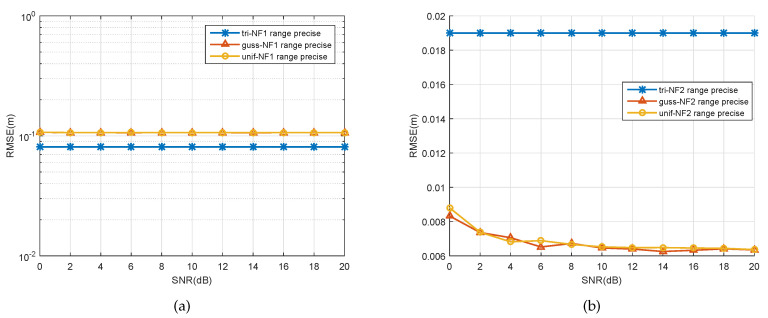
RMSE of the range estimates of two signals versus SNR. (**a**) First spatially distributed source, (**b**) second spatially distributed source.

**Figure 8:**
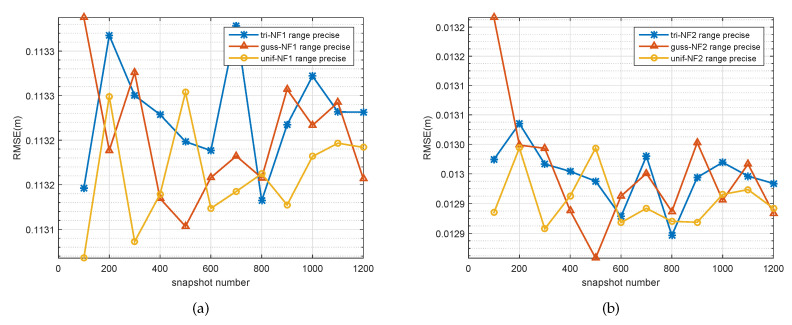
RMSE of the range estimates of two signals versus snapshot. (**a**) First spatially distributed source with three kinds of PDF, (**b**) second spatially distributed source with three kinds of PDF.

**Figure 9:**
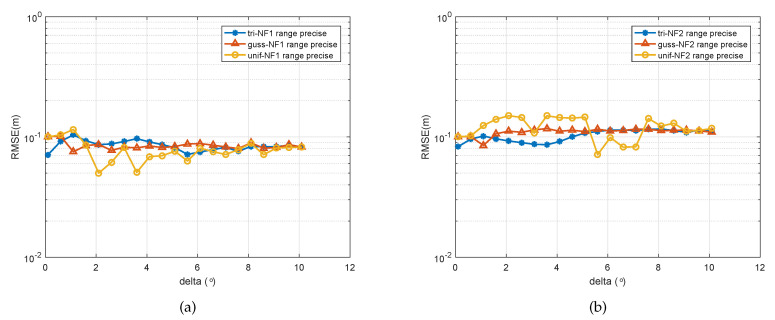
RMSE of the range estimates of two signals versus parameter ∇. (**a**) First spatially distributed source with three types of PDF, (**b**) second spatially distributed source with three types of PDF.

So far, various cases of DOA estimation were discussed, except for range estimation for the spatially distributed source. From Figure 7, Figure 8, Figure 9, we can see that the range estimations had an irregular influence on the SNR, snapshot, and ∇. The signal model in this paper is based on an assumption that the angle follows some PDF, which is a pragmatic approach to real applications to near-filed sources. A change of angular information will influence the range of the near-field source. Due to the fact that the variation between these two parameters is nonlinear, coupled with the PDF of the source, it is hard to find rules for the range estimation; although the performance of range estimation is satisfactory, we still have a very accurate range estimator (we can see that all the errors for the range estimator were under the orders of magnitude $10^{-1}$).

## 4. Conclusions

The near-field localization problem of non-circular distributed sources was addressed in this paper. The traditional use for distributed source processing is only for far-field sources, and it generally needs a 2D searching method or omits the influence of angular spread for the DOA estimation. In order to improve estimation performance, the non-circularity of signals was taken into account. By carefully examining the structure of the steering vector, we decoupled the DOA and range from the non-circular phase and angular spread, and obtained the estimates by the RARE principle in sequence. The proposed method exhibited satisfactory localization performance for near-field non-circular sources.
